# Single-cell and bulk transcriptomic analyses define an IFI27-centered interferon-response epithelial state in bladder cancer and its transcriptional response to irradiation

**DOI:** 10.3389/fcell.2026.1920354

**Published:** 2026-09-11

**Authors:** Yang Ji, Yangyang Zhang, Xiaodong Liu, Zhuoyuan Lv, Yakai Wang, Shixuan Wu, Lei Wang

**Affiliations:** 1 Urology Department, East Hospital Area, Xinxiang Central Hospital, Xinxiang, Henan, China; 2 Department of Urology, Xinxiang Central Hospital, Xinxiang, Henan, China; 3 The Fourth Clinical College of Henan Medical University, Henan Medical University, Xinxiang, Henan, China

**Keywords:** antigen presentation, bladder cancer, interferon response, single-cell RNA sequencing, tumor microenvironment

## Abstract

**Background:**

Interferon-responsive tumor-cell states can simultaneously increase immune visibility and induce immune-regulatory programs, yet their epithelial-cell distribution and treatment responsiveness in bladder cancer remain incompletely defined. We sought to resolve a reproducible epithelial interferon-response state across patients, characterize its immune context, and determine whether its transcriptional components respond to ionizing radiation *in vitro*.

**Methods:**

Single-cell RNA sequencing data from seven primary bladder tumors and one adjacent normal sample were reanalyzed with uniform quality control and doublet removal. After broad-lineage annotation, 29,777 identity-gated tumor epithelial cells were reclustered and analyzed by repeated non-negative matrix factorization across ranks and random seeds. An NMF-informed three-gene interferon-response score based on IFI27, ISG15, and IFI6 was used to define patient-internal upper- and lower-quartile states. Differential expression was assessed by patient-blocked pseudobulk edgeR models, followed by Hallmark, Gene Ontology, and Reactome enrichment, leave-one-patient-out analyses, immune-feature scoring, balanced CellChat analysis, and TCGA-BLCA corroboration. T24 bladder cancer cells were exposed to 0, 2, 4, or 8 Gy, and selected transcripts were quantified by qRT-PCR 24 h later.

**Results:**

The final atlas comprised 39,224 cells assigned to six broad lineages. Repeated NMF reproducibly identified an IFI27-centered epithelial program, and the corresponding score showed marked interpatient variation while remaining detectable across all seven tumors. Patient-blocked analysis identified 179 genes with false-discovery rate <0.05 and absolute log2FC ≥ 0.5; IFIT1, CXCL10, IFI44L, IFITM1, HLA-B, B2M, CD74, IFI6, and ISG15 were representative upregulated genes. IFN-response-score-high epithelial cells showed strong enrichment of interferon-gamma response, interferon-alpha response, inflammatory response, IL6-JAK-STAT3 signaling, antigen processing and presentation, and chemokine-response programs. MHC-I, MHC-II, CXCL9/10/11-related, and immune-regulatory ligand scores increased in all seven patient-paired comparisons, whereas CD274 itself remained sparsely detected and was not stably associated with the continuous score. Balanced pooled CellChat analyses prioritized relatively enriched MDK-SDC1, MDK-SDC2, and MDK-ITGA6/ITGB1 candidate interactions. TCGA-BLCA analyses corroborated the broader interferon and antigen-presentation context at gene and pathway levels. Irradiation induced dose-associated increases in IFI27, ISG15, IFI6, IFIT1, CXCL10, and IFITM1 transcripts, accompanied by increases in CD274, VIM, and SNAI1 and a decrease in CDH1.

**Conclusion:**

Bladder tumors contain a reproducible IFI27-centered epithelial interferon-response state with coordinated antigen-presentation, chemokine-related, and selected immune-regulatory transcriptional features. Its molecular context is supported across patients and in bulk transcriptomes, while irradiation-associated qRT-PCR changes suggest that components of the program are treatment responsive. These findings define a hypothesis-generating epithelial immune state and candidate communication axes for future spatial, protein-level, and functional validation.

## 1 Introduction

Bladder cancer remains a major global health burden, with more than 600,000 new cases and approximately 220,000 deaths estimated worldwide in 2022 (Bray et al., 2024). Urothelial carcinoma accounts for most cases and spans biologically distinct non-muscle-invasive and muscle-invasive forms, with substantial variation in recurrence, progression, treatment response, and survival (Lenis et al., 2020). Large-scale molecular studies have further shown that bladder tumors comprise multiple genomic and transcriptomic classes with divergent epithelial differentiation, immune infiltration, stromal content, and clinical behavior (Robertson et al., 2017; Kamoun et al., 2020). This heterogeneity creates a strong rationale for resolving tumor-cell states at single-cell resolution rather than treating bulk tumors as uniform biological entities.

Interferon signaling is a central component of tumor-immune biology. Radiation and other forms of cellular stress can promote cytosolic nucleic-acid sensing, type I interferon production, antigen processing, chemokine expression, and immune-cell recruitment (Demaria et al., 2016; Sharabi et al., 2015; Vanpouille-Box et al., 2017; Yang et al., 2021; Wang et al., 2023). At the same time, sustained tumor-intrinsic interferon signaling can induce adaptive immune-regulatory programs and contribute to treatment resistance through multiple inhibitory pathways (Benci et al., 2016). Clinically derived interferon-gamma-related gene-expression profiles often integrate antigen presentation, chemokine expression, cytotoxic immune activity, and immune-regulatory ligands, underscoring the dual rather than uniformly beneficial nature of this biology (Ayers et al., 2017). Accordingly, an epithelial interferon-response state cannot be interpreted solely as an indicator of antitumor immunity or immune escape; its cellular context and accompanying gene programs must be defined directly.

Single-cell RNA sequencing has revealed extensive cellular and transcriptional diversity in bladder cancer, including heterogeneous malignant epithelial populations, inflammatory and myofibroblastic stromal states, diverse myeloid programs, and treatment-associated remodeling (Lai et al., 2021; Chen et al., 2020; Ma et al., 2022; Sfakianos et al., 2020). These studies also highlight a recurring analytical challenge: patient-specific variation can dominate unsupervised clusters and can generate misleading differential-expression results when cells are treated as independent biological replicates. Robust inference therefore requires explicit retention of the patient level, careful exclusion of doublets and lineage contamination, and assessment of program stability across analytical settings (Papalexi and Satija, 2018; Stuart and Satija, 2019).

In this study, we reanalyzed a multi-patient bladder cancer single-cell dataset using uniform quality control, identity gating, repeated non-negative matrix factorization (NMF), patient-internal state definition, and patient-blocked pseudobulk statistics. Unsupervised NMF reproducibly identified an IFI27-centered interferon-response program. We characterized this state at gene, pathway, immune-feature, and candidate cell-communication levels, examined its robustness across patients, corroborated its broader context in TCGA-BLCA, and evaluated irradiation-associated transcriptional responses in T24 bladder cancer cells. The study was designed to define a reproducible transcriptomic state and its candidate context, rather than to infer functional immune activity or radioresistance from expression data alone.

## 2 Materials and methods

### 2.1 Study design and data sources

Publicly available single-cell RNA sequencing data were obtained from the Gene Expression Omnibus dataset GSE135337, which includes primary bladder tumor and adjacent bladder tissue specimens generated on the 10x Genomics platform (Lai et al., 2021). Seven tumor samples (BC1-BC7) and one adjacent normal sample (BCN) were included. The adjacent normal sample was used only as a descriptive reference because it represented a single biological specimen; no inferential tumor-versus-normal differential-expression analysis was performed. TCGA Bladder Urothelial Carcinoma (TCGA-BLCA) RNA-sequencing and clinical data were obtained from the Genomic Data Commons. The analytical workflow focused on tumor-internal epithelial heterogeneity, patient-blocked state comparisons, cross-platform corroboration, and *in vitro* irradiation-associated qRT-PCR.

### 2.2 Single-cell quality control, normalization, and integration

Genes detected in fewer than 3 cells were removed. Cells were retained when they contained 500–6,000 detected genes and a mitochondrial transcript fraction below 10%; outlying library sizes were reviewed within each sample. Predicted doublets were identified with scDblFinder and excluded (Germain et al., 2021). After quality control and doublet removal, 39,224 cells were retained. Counts were normalized to 10,000 transcripts per cell and log transformed. Highly variable genes were selected, principal component analysis was performed, and Harmony was used to reduce sample-associated technical variation in the low-dimensional embedding (Korsunsky et al., 2019). Harmony-corrected components were used for neighborhood construction, clustering, and UMAP visualization only; expression summaries, module scores, NMF, and pseudobulk analyses used nonnegative normalized expression values or raw UMI counts, as appropriate.

### 2.3 Broad-lineage annotation and epithelial identity filtering

Broad lineages were annotated by combined marker expression rather than single genes. Epithelial cells were identified by EPCAM and keratin genes including KRT7, KRT8, KRT18, KRT19, and KRT20; fibroblasts by COL1A1, COL1A2, COL3A1, DCN, and LUM; myeloid cells by LST1, TYROBP, FCER1G, CTSS, and CD68; T/NK cells by CD3D, CD3E, TRBC1, NKG7, and GNLY; endothelial cells by PECAM1, VWF, EMCN, and KDR; and perivascular cells by RGS5, ACTA2, MCAM, and CSPG4. This yielded six broad lineages.

The initial tumor-derived epithelial candidate compartment contained 32,734 cells. To reduce residual immune, stromal, endothelial, and doublet contamination, a conservative identity gate was applied. Retained cells expressed at least two epithelial markers among EPCAM, KRT7, KRT8, KRT18, KRT19, UPK1A, and UPK2; had no detected immune-lineage markers among PTPRC, LST1, and TYROBP; had no detected endothelial markers among PECAM1 and VWF; and expressed fewer than two fibroblast markers among COL1A1, COL1A2, DCN, and LUM. The final epithelial analysis set comprised 29,777 cells. Highly variable gene selection, PCA, Harmony integration, neighborhood construction, clustering, and UMAP were repeated on this filtered compartment, yielding 16 epithelial subclusters labeled Epi-1 to Epi-16.

### 2.4 NMF program discovery and IFN-response state definition

NMF was applied to the nonnegative log-normalized epithelial expression matrix to identify recurrent cellular programs (Kotliar et al., 2019). Ranks 6, 8, and 10 were each evaluated with five random seeds, generating 15 runs. Programs were matched across runs by cosine similarity of gene loadings, and stability was summarized by loading-vector cosine similarity, top-50-gene Jaccard similarity, and anchor-gene rank consistency. A reproducible IFI27-centered program was identified in all 15 runs; IFI27, ISG15, and IFI6 entered the top 100 loading genes in every run.

For an interpretable and reproducible state score, the mean log-normalized expression of IFI27, ISG15, and IFI6 was calculated for each epithelial cell and termed the NMF-informed three-gene IFN-response score. Within each patient, cells in the upper score quartile were defined as IFN-response-score-high (IFN-RS-high), cells in the lower quartile as IFN-response-score-low (IFN-RS-low), and the middle 50% as an intermediate group excluded from high-versus-low differential-expression analysis. Patient-internal thresholds ensured that every tumor contributed to the state comparison despite marked differences in absolute score distributions.

### 2.5 Patient-blocked pseudobulk differential expression and robustness analyses

Because cells from the same patient are not independent biological replicates, raw UMI counts were aggregated by patient and IFN-response state to generate one pseudobulk profile per patient-state combination (Squair et al., 2021). Differential expression was analyzed with edgeR using a patient-blocked design (∼ patient + state), trimmed mean of M values normalization, robust dispersion estimation, and quasi-likelihood testing (Lun et al., 2016). Genes were considered differentially expressed when the Benjamini–Hochberg false-discovery rate (FDR) was below 0.05 and the absolute log2 fold change was at least 0.5. Patient-specific high-minus-low logCPM differences were treated as descriptive effects; only the patient-blocked model provided inferential effect estimates and confidence intervals.

Robustness was examined by leave-one-patient-out analysis. The pseudobulk model and selected enrichment analyses were repeated seven times, each time excluding one patient. Stability was assessed from the direction and range of gene-level effects and normalized enrichment scores. NMF stability across ranks and seeds was evaluated separately from differential-expression robustness.

### 2.6 Gene-set enrichment analysis

The complete patient-blocked gene ranking was analyzed by preranked gene-set enrichment analysis using Hallmark gene sets, Gene Ontology Biological Process collections, and Reactome pathways (Sub et al., 2005; Liberzon et al., 2015). Normalized enrichment scores (NES), nominal P values, and FDR values were reported. Hallmark analyses included interferon-gamma response, interferon-alpha response, inflammatory response, complement, IL6-JAK-STAT3 signaling, TNF-alpha signaling via NF-kappaB, apoptosis, epithelial-mesenchymal transition, E2F targets, G2M checkpoint, and MYC targets. Leading-edge genes were summarized at the patient-pseudobulk level.

### 2.7 Immune-effector and immune-regulatory transcriptional features

Four epithelial immune-feature scores were calculated from predefined gene sets that excluded IFI27, ISG15, and IFI6: (1) MHC-I antigen processing and presentation (HLA-A, HLA-B, HLA-C, B2M, TAP1, TAP2, PSMB8, PSMB9, and NLRC5); (2) MHC-II-related antigen presentation (CD74, HLA-DRA, HLA-DRB1, HLA-DPA1, HLA-DPB1, and CIITA); (3) CXCL9/10/11 T-cell-recruitment-related chemokines; and (4) immune-regulatory ligands (HLA-E, LGALS9, PVR, NECTIN2/PVRL2, and CD274). Module scores were averaged within each patient-state combination and compared with two-sided exact paired Wilcoxon tests, with BH correction across the four modules.

To evaluate continuous relationships, cells were divided into 10 patient-internal score deciles. Expression and module scores were aggregated to 70 patient-by-decile observations. Standardized mixed-effects models included score decile as a fixed effect and patient as a random intercept. Gene-level patient-pseudobulk high-minus-low effects were displayed separately to identify cross-patient consistency. Low-detection genes were not assigned zero effect when stable pseudobulk estimates were unavailable.

### 2.8 Candidate cell-cell communication analysis

CellChat was used to infer candidate ligand-receptor communication from expression data and curated multimeric interaction knowledge (Jin et al., 2021). To reduce the influence of unequal epithelial group size, 700 IFN-RS-high and 700 IFN-RS-low epithelial cells were sampled with patient balance. Tumor-derived receiver populations comprised 438 myeloid cells, 220 T/NK cells, 108 fibroblasts, and 70 endothelial cells. Population-size weighting was disabled. High and low states were evaluated using the same ligand-receptor universe; raw communication probabilities were retained even when a state-specific permutation threshold was not met, preventing nonsignificant probabilities from being treated as zero.

Communication results were interpreted as pooled candidate interactions rather than patient-level statistical comparisons. Resampling stability was examined in 20 patient-balanced iterations, each drawing 80 IFN-RS-high and 80 IFN-RS-low epithelial cells per patient where available. The frequency and median high-minus-low probability difference were summarized for selected interactions. Because receiver-cell numbers were limited and patient-specific networks were not independently estimated, no inferred interaction was considered mechanistically validated.

### 2.9 TCGA-BLCA bulk-transcriptomic corroboration

For TCGA-BLCA primary tumors, sample-wise standardized expression values for IFI27, ISG15, and IFI6 were averaged to generate a three-gene IFN-response score. Score-ordered heatmaps displayed independent interferon-response genes, antigen-processing and presentation genes, and immune-regulatory ligands. Available pathologic stage, consensus molecular subtype, tumor-purity estimates, and immune scores were included as annotations. Consensus subtypes were interpreted according to the established six-class MIBC framework (Kamoun et al., 2020).

For gene-level corroboration, tumors in the upper and lower score quartiles were compared while the middle 50% were excluded. Models included tumor purity as a covariate. Defining genes were excluded from cross-platform concordance analysis. Independent Hallmark interferon-gamma, Hallmark interferon-alpha, and Reactome antigen-processing scores were computed after removing IFI27, ISG15, and IFI6. Gene-level effects and Hallmark NES values were compared with the patient-blocked single-cell results. MCP-counter was used to estimate immune and stromal features from bulk transcriptomes (Becht et al., 2016), and crude as well as tumor-purity-adjusted associations were reported. Clinical analyses used continuous standardized scores in predefined models; subgroup plots were descriptive unless supported by patient-level inference.

### 2.10 Cell culture, irradiation, and qRT-PCR

The human T24 bladder cancer cell line (ATCC HTB-4) was maintained in McCoy’s 5 A medium supplemented with 10% fetal bovine serum and 1% penicillin-streptomycin at 37 °C in 5% CO_2_. Cells were authenticated by short tandem repeat profiling and confirmed to be *mycoplasma* negative before use. Cells were seeded at 3 × 10^5^ cells per well in six-well plates and allowed to adhere for 24 h. Single-fraction irradiation was delivered at 0, 2, 4, or 8 Gy using 6-MV photons at 2 Gy/min. Total RNA was collected 24 h after irradiation.

RNA was extracted with TRIzol, and 1 μg was reverse transcribed. qRT-PCR was performed with SYBR Green chemistry on an ABI 7500 system. The primary interferon-response panel comprised IFI27, ISG15, IFI6, IFIT1, CXCL10, and IFITM1. CD274, VIM, SNAI1, and CDH1 were assessed as auxiliary immune-regulatory and EMT-related transcripts. GAPDH served as the endogenous control. Relative expression was calculated by the 2^−ΔΔCt method with the 0-Gy group as reference. Statistical tests were performed on ΔCt values, whereas fold changes were used for presentation. All experiments included three independent biological replicates.

### 2.11 Statistical analysis

All tests were two sided. P values were adjusted by the Benjamini–Hochberg method where multiple hypotheses were evaluated, and FDR <0.05 was considered statistically significant. Patient-paired comparisons used exact Wilcoxon tests. Mixed-effects models were fitted to patient-by-decile aggregates. qRT-PCR dose groups were compared by one-way analysis of variance with Dunnett adjustment against 0 Gy when distributional assumptions were met; otherwise, the corresponding rank-based procedure was used. Correlations were assessed by Spearman or Pearson methods as specified for each analysis. Analyses emphasized effect size, cross-patient direction, and sensitivity to patient exclusion in addition to nominal significance.

## 3 Results

### 3.1 Multi-patient single-cell landscape of bladder cancer

**FIGURE 1:**
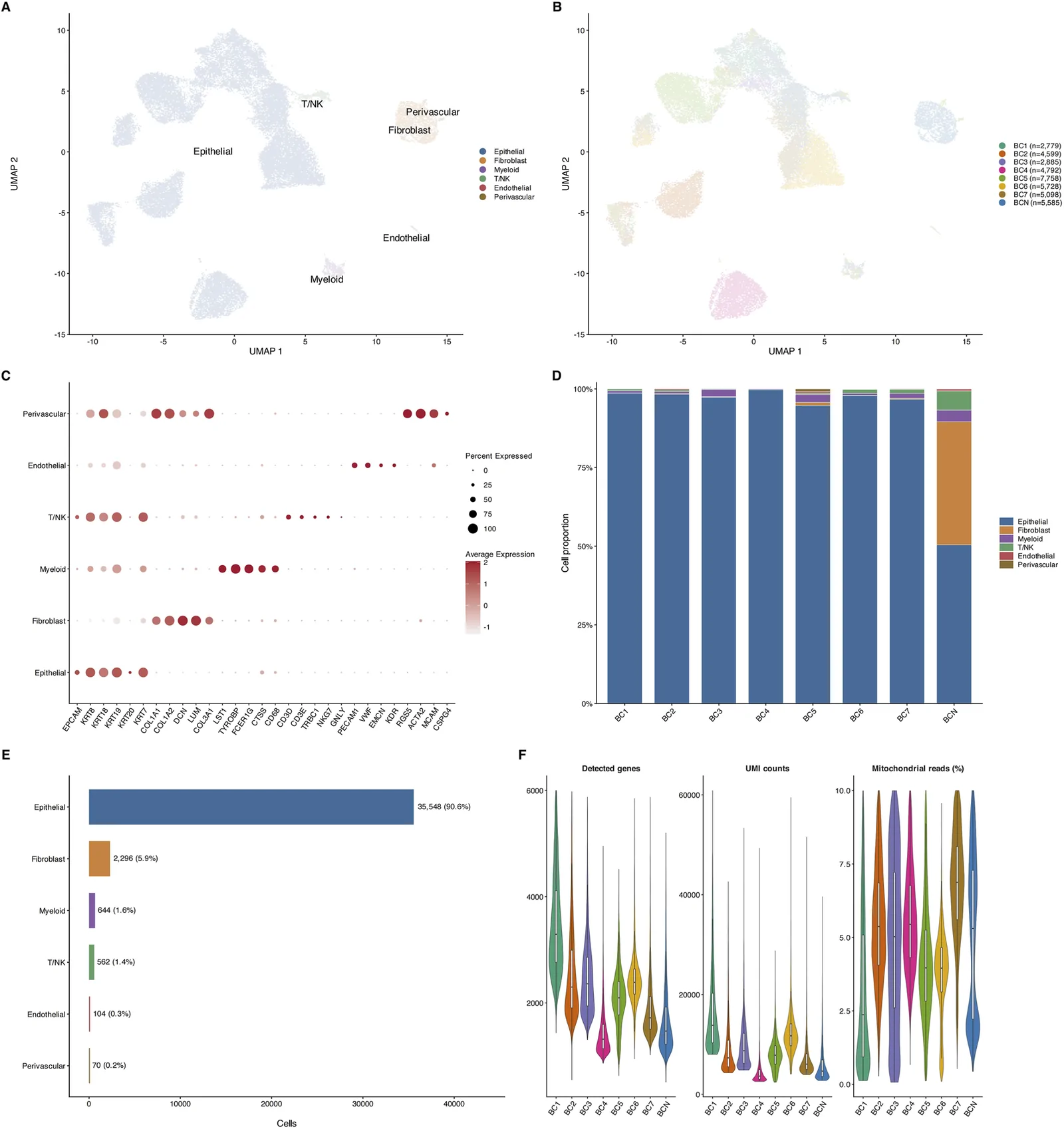
Multi-patient single-cell landscape of bladder cancer. **(A)** UMAP of 39,224 quality-controlled cells colored by six broad lineages. **(B)** The same UMAP colored by sample of origin; sample-specific cell numbers are indicated. **(C)** Dot plot of canonical marker genes supporting epithelial, fibroblast, myeloid, T/NK, endothelial, and perivascular annotations. Dot size represents the fraction of expressing cells and color represents mean log-normalized expression. **(D)** Relative lineage composition of BC1-BC7 and BCN. The single adjacent normal sample was included for descriptive reference only. **(E)** Total cell number and percentage for each lineage. **(F)** Sample-level distributions of detected genes, UMI counts, and mitochondrial transcript fractions after uniform quality control and scDblFinder-based doublet removal.

Uniform quality control and scDblFinder-based doublet removal retained 39,224 cells across eight specimens: BC1, 2,779; BC2, 4,599; BC3, 2,885; BC4, 4,792; BC5, 7,758; BC6, 5,728; BC7, 5,098; and BCN, 5,585. UMAP and combined marker expression supported six broad lineages: 35,548 epithelial cells (90.6%), 2,296 fibroblasts (5.9%), 644 myeloid cells (1.6%), 562 T/NK cells (1.4%), 104 endothelial cells (0.3%), and 70 perivascular cells (0.2%) (Figures 1A–C).

Tumor samples were dominated by epithelial cells, which accounted for 94.7%–99.7% of cells in each of the seven tumors. In contrast, BCN contained 50.4% epithelial cells, 39.2% fibroblasts, 6.12% T/NK cells, and 3.69% myeloid cells (Figures 1D,E). Because BCN represented a single specimen, these differences were treated as descriptive composition rather than a basis for formal group inference. Median detected genes ranged from 1,316 to 3,292 per sample, median UMI counts from 3,640 to 13,812, and median mitochondrial fractions from 2.37% to 6.88% (Figure 1F). Sample-colored UMAPs showed marked patient-specific clustering, establishing the need to retain patient structure in all downstream comparisons.

### 3.2 Epithelial heterogeneity and identification of an IFI27-centered NMF program

**FIGURE 2:**
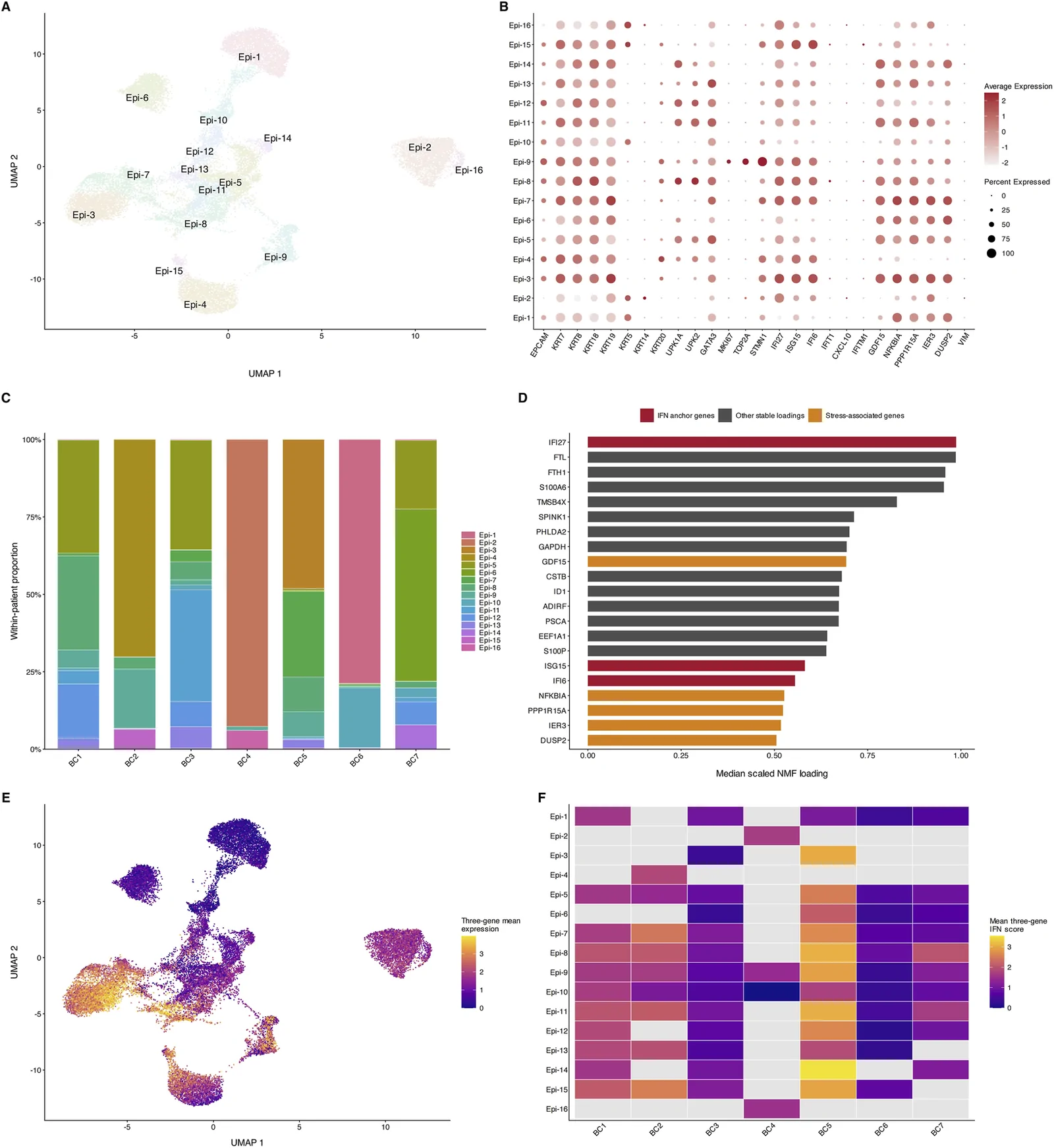
Tumor epithelial heterogeneity and identification of an IFI27-centered NMF program. **(A)** UMAP of 29,777 identity-gated tumor epithelial cells reclustered into 16 neutral subclusters. **(B)** Dot plot of epithelial differentiation, proliferation, interferon-response, stress-response, and auxiliary markers across subclusters. **(C)** Patient-specific epithelial subcluster composition. **(D)** Stable gene loadings of the matched IFI27-centered program across NMF ranks 6, 8, and 10 and five random seeds per rank. **(E)** UMAP distribution of the NMF-informed three-gene IFN-response score, defined as the mean log-normalized expression of IFI27, ISG15, and IFI6. **(F)** Heatmap of mean score across patient-by-subcluster combinations; gray indicates combinations with no retained cells.

Conservative epithelial identity filtering reduced 32,734 initial tumor epithelial candidates to 29,777 cells. Recalculation of variable genes, PCA, Harmony integration, clustering, and UMAP within this filtered set yielded 16 epithelial subclusters (Figure 2A). Most clusters retained EPCAM and KRT7/8/18/19 expression. Epi-7 was distinguished by MKI67, TOP2A, and STMN1, consistent with a proliferative state, whereas interferon-associated genes were distributed across multiple clusters rather than confined to a single discrete population (Figure 2B). Cluster composition was strongly patient dependent: the largest clusters were Epi-5 in BC1 (36.5%), Epi-4 in BC2 (70.2%), Epi-11 in BC3 (36.1%), Epi-2 in BC4 (92.7%), Epi-3 in BC5 (48.1%), Epi-1 in BC6 (78.8%), and Epi-6 in BC7 (55.6%) (Figure 2C).

Unsupervised NMF reproducibly identified an IFI27-centered program. Across 15 runs spanning ranks 6, 8, and 10, median normalized loadings were 0.987 for IFI27, 0.581 for ISG15, and 0.555 for IFI6; all three genes entered the matched program’s top 100 loadings in 15 of 15 runs (Figure 2D). The NMF-informed three-gene IFN-response score formed a continuous gradient across the epithelial UMAP (Figure 2E). Program activity reflected both cluster identity and patient origin: BC5 showed broadly elevated scores across several clusters, but high-score regions were also detectable in other tumors, including Epi-8 in BC1, BC2, and BC7 (Figure 2F).

### 3.3 Patient-blocked analysis defines a broader IFN-response epithelial state

**FIGURE 3:**
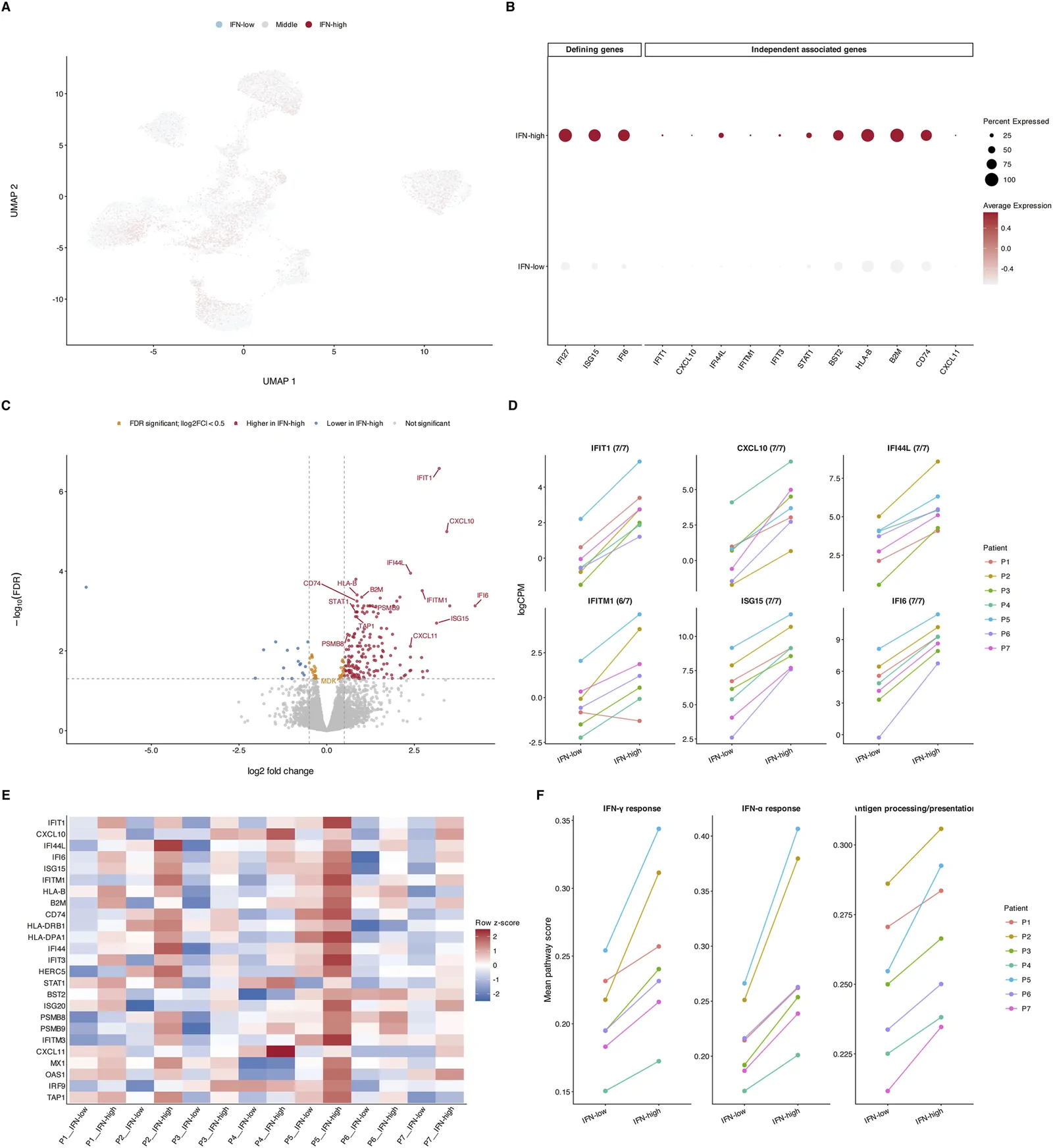
IFN-response-score-high epithelial state and patient-blocked differential expression. **(A)** UMAP showing patient-internal IFN-RS-low, middle, and IFN-RS-high states. **(B)** Dot plot separating score-defining genes from independent associated genes. **(C)** Volcano plot from the patient-blocked pseudobulk edgeR model; MDK is annotated as FDR significant but below the predefined effect-size threshold. **(D)** Descriptive patient-specific high-minus-low effects for selected genes, together with the patient-blocked overall effect. Single-patient effects are shown without confidence intervals. **(E)** Patient-paired pseudobulk heatmap of representative differentially expressed genes. **(F)** Patient-paired validation of extended interferon-gamma, interferon-alpha, and antigen-processing scores after removal of IFI27, ISG15, and IFI6.

Within each tumor, the upper and lower quartiles of the three-gene score defined IFN-RS-high and IFN-RS-low states, respectively (Figure 3A). As expected, the defining genes IFI27, ISG15, and IFI6 were enriched in the high state. More importantly, independent genes not used to construct the score-including IFIT1, CXCL10, IFI44L, IFITM1, IFIT3, STAT1, BST2, HLA-B, B2M, CD74, and CXCL11-also showed coordinated increases (Figure 3B).

Patient-blocked pseudobulk edgeR analysis identified 179 genes with FDR <0.05 and absolute log2FC ≥ 0.5 (Figure 3C). Representative effects included IFIT1 (log2 fold change 3.210, FDR 2.66 × 10^−7^), CXCL10 (3.426, 1.02 × 10^-5^), IFI44 L (2.390, 1.12 × 10^−4^), IFITM1 (2.724, 3.08 × 10^−4^), HLA-B (0.864, 3.94 × 10^−4^), B2M (1.000, 4.48 × 10^−4^), CD74 (0.861, 5.64 × 10^−4^), IFI6 (4.235, 7.42 × 10^−4^), and ISG15 (3.134, 0.00201). MDK met the FDR criterion but had a log2 fold change of 0.470 and therefore did not meet the predefined effect-size threshold.

IFIT1, CXCL10, IFI44L, ISG15, and IFI6 were higher in IFN-RS-high than IFN-RS-low pseudobulks in all seven patients, whereas IFITM1 showed the same direction in six of seven patients (Figure 3D). A paired patient-level heatmap confirmed broadly concordant elevation of interferon-stimulated, antigen-processing, and presentation-associated genes, while preserving interpatient differences in magnitude (Figure 3E). To avoid circular confirmation, extended Hallmark interferon-gamma, Hallmark interferon-alpha, and Reactome antigen-processing scores were recalculated after removing IFI27, ISG15, and IFI6. All three independent scores were higher in IFN-RS-high cells in seven of seven patients, with median high-minus-low differences of 0.0365, 0.0518, and 0.0164, respectively (Figure 3F).

### 3.4 IFN-RS-high epithelial cells are enriched for interferon, inflammatory, and chemokine-response programs

**FIGURE 4:**
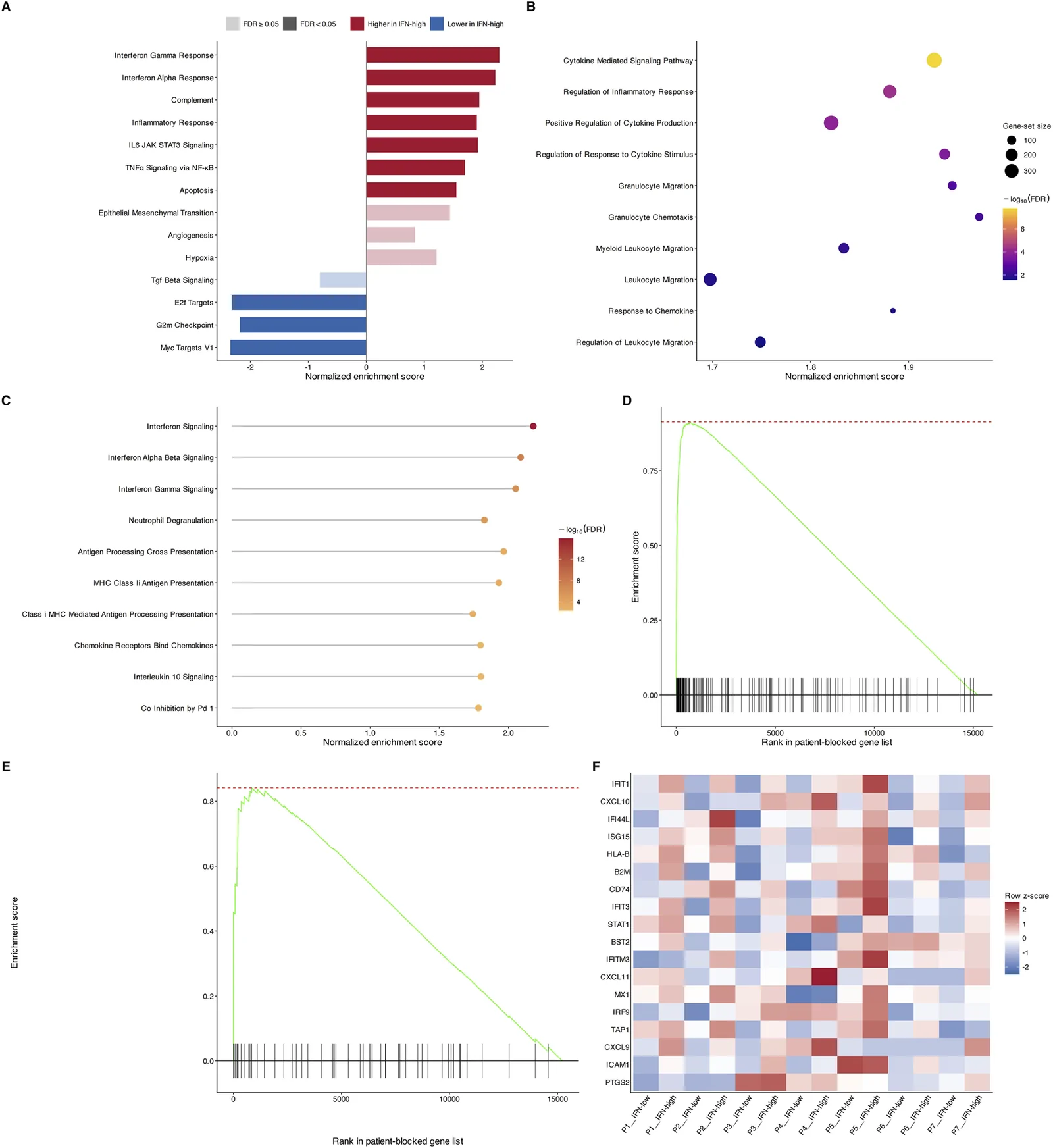
Functional enrichment of the IFN-response-score-high epithelial state. **(A)** Hallmark FGSEA overview showing positively and negatively enriched pathways; EMT is marked as a nonsignificant trend. **(B)** GO Biological Process enrichment of cytokine, inflammatory, chemokine-response, and leukocyte-migration programs. **(C)** Reactome enrichment lollipop plot. **(D)** Interferon-gamma-response enrichment curve. **(E)** Response-to-chemokine enrichment curve. **(F)** Patient-paired leading-edge gene heatmap. NES, normalized enrichment score; FDR, false-discovery rate.

Hallmark FGSEA revealed strong positive enrichment of interferon-gamma response (NES 2.295, FDR 1.58 × 10^−24^), interferon-alpha response (NES 2.226, FDR 3.45 × 10^−16^), complement (NES 1.948, FDR 2.36 × 10^−6^), inflammatory response (NES 1.906, FDR 9.21 × 10^−6^), IL6-JAK-STAT3 signaling (NES 1.923, FDR 2.19 × 10^-5^), TNF-alpha signaling via NF-kappaB (NES 1.703, FDR 0.00276), and apoptosis (NES 1.553, FDR 0.0132) in IFN-RS-high epithelial cells (Figure 4A). E2F targets, G2M checkpoint, and MYC targets V1 were negatively enriched, suggesting lower proliferative transcriptional activity in the high-score state. EMT showed a positive but nonsignificant trend (NES 1.442, FDR 0.0918) and was not considered a defining feature.

GO Biological Process analysis supported cytokine-mediated signaling (NES 1.926, FDR 1.65 × 10^−8^), inflammatory-response regulation (NES 1.881, FDR 4.59 × 10^-5^), granulocyte migration (NES 1.945, FDR 0.00190), granulocyte chemotaxis (NES 1.972, FDR 0.00351), myeloid leukocyte migration (NES 1.834, FDR 0.00853), leukocyte migration (NES 1.697, FDR 0.0192), and response to chemokine (NES 1.884, FDR 0.0202) (Figure 4B). Reactome independently identified interferon signaling (NES 2.179, FDR 1.47 × 10^−16^), interferon-alpha/beta signaling (NES 2.087, FDR 2.45 × 10^−8^), interferon-gamma signaling (NES 2.052, FDR 7.41 × 10^−7^), neutrophil degranulation (NES 1.826, FDR 5.33 × 10^−6^), antigen processing and presentation, and chemokine-receptor binding (NES 1.798, FDR 0.00322) (Figure 4C). Enrichment curves and leading-edge heatmaps confirmed that these results were driven by coordinated changes in multiple genes across patients rather than a single anchor gene (Figures 4D–F).

### 3.5 The IFI27-centered program is stable across patients and NMF settings

**FIGURE 5:**
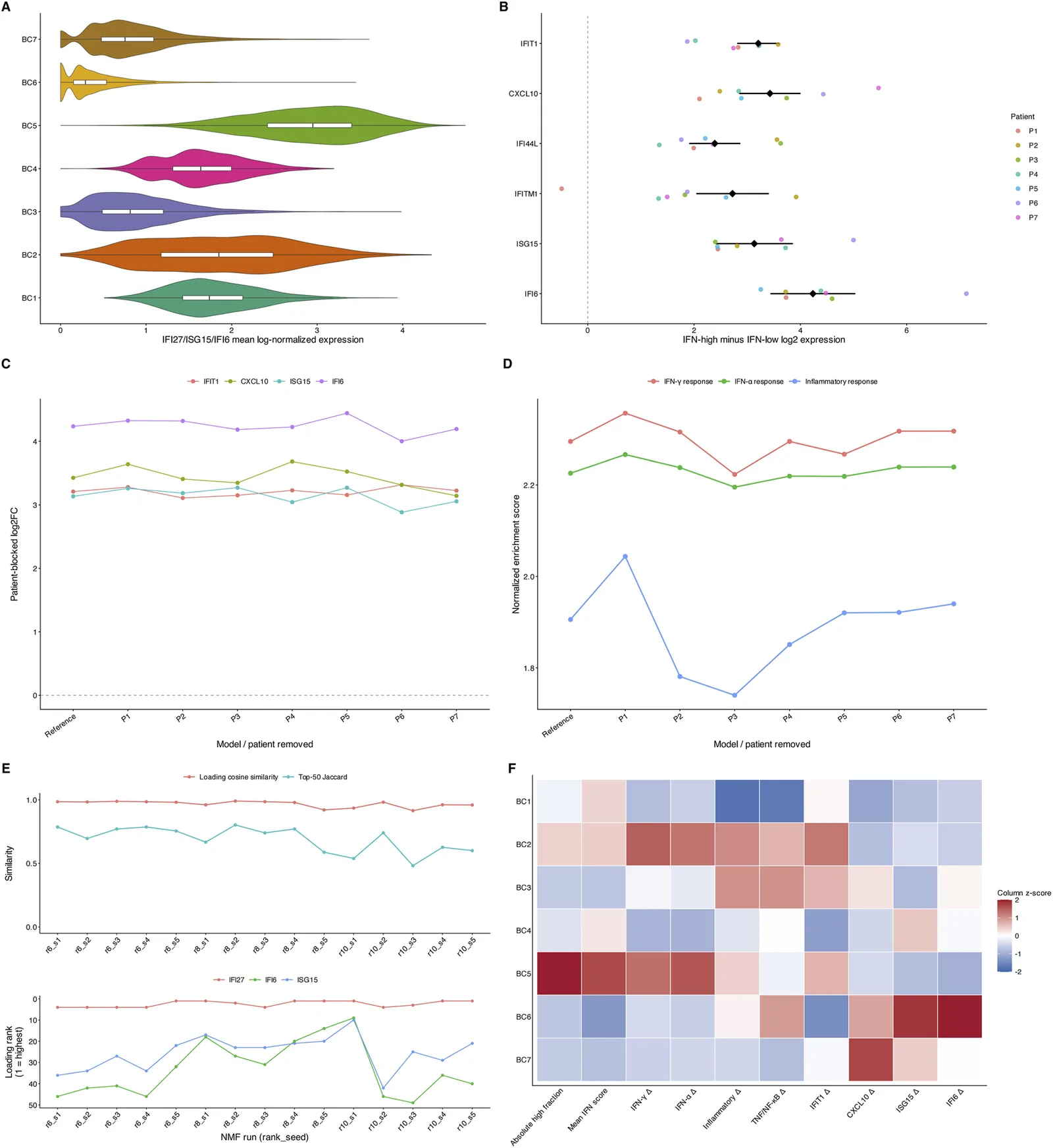
Cross-patient robustness of the interferon-response epithelial program. **(A)** Distribution of the NMF-informed three-gene IFN-response score in each tumor. **(B)** Patient-specific descriptive effects and patient-blocked overall effects for selected genes. **(C)** Leave-one-patient-out gene-level sensitivity analysis. **(D)** Leave-one-patient-out pathway-level sensitivity analysis. **(E)** NMF stability across ranks and random seeds, including loading-vector cosine similarity, top-50-gene Jaccard similarity, and anchor-gene loading ranks. **(F)** Integrated patient-level heatmap summarizing absolute state abundance, independent pathway-score differences, and selected gene effects.

Absolute three-gene IFN-response scores differed markedly among tumors, with BC5 showing the highest overall distribution and BC6 the lowest (Figure 5A). Nevertheless, patient-internal high-minus-low effects remained directionally stable. Median descriptive effects were 2.831 for IFIT1, 2.891 for CXCL10, 2.211 for IFI44L, 2.811 for ISG15, 4.386 for IFI6, and 1.829 for IFITM1 (Figure 5B).

Leave-one-patient-out models retained positive effects for IFIT1, CXCL10, ISG15, and IFI6 regardless of which patient was removed (Figure 5C). Pathway-level robustness was similar: across the complete model and seven leave-one-out analyses, interferon-gamma response NES ranged from 2.223 to 2.357, interferon-alpha response from 2.195 to 2.266, and inflammatory response from 1.740 to 2.044; all corresponding FDR values remained below 0.05 (Figure 5D).

The matched NMF program was also stable across ranks and random seeds. Median loading-vector cosine similarity ranged from 0.914 to 0.990, and median top-50-gene Jaccard similarity ranged from 0.482 to 0.802. IFI27, ISG15, and IFI6 entered the top 100 genes in every run (Figure 5E). A patient-level summary showed that the core interferon direction was consistent despite substantial heterogeneity in absolute high-state abundance and in auxiliary inflammatory and NF-kappaB features (Figure 5F).

### 3.6 Balanced pooled communication analysis prioritizes MDK-related candidate interactions

**FIGURE 6:**
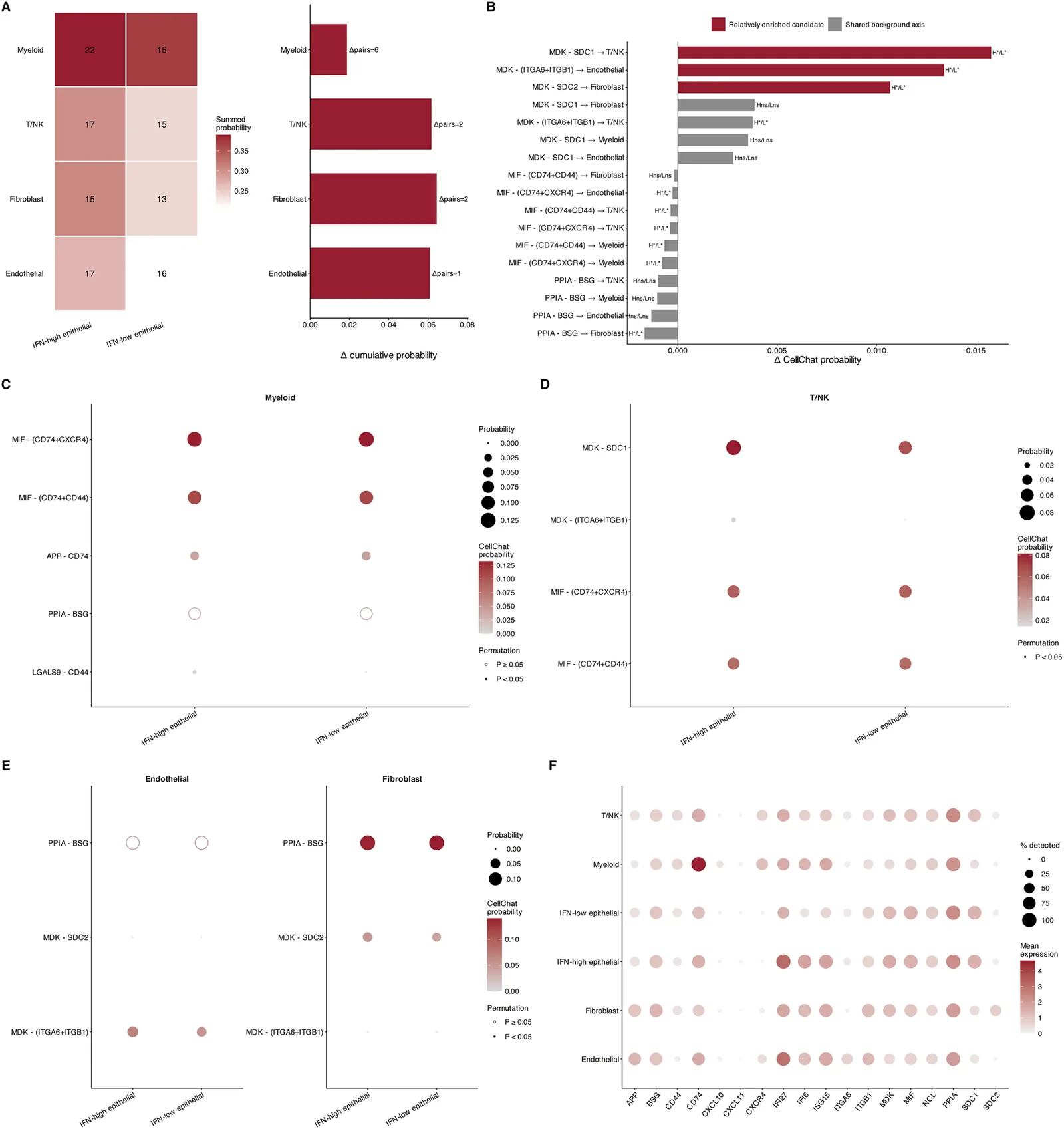
Candidate communication profiles of IFN-response-score-high epithelial cells. **(A)** Balanced pooled comparison of supported outgoing ligand-receptor pair counts and cumulative communication probabilities toward myeloid, T/NK, fibroblast, and endothelial cells. **(B)** High-minus-low probability differences calculated from a common ligand-receptor universe, with state-specific permutation status indicated. **(C)** Candidate epithelial-to-myeloid interactions; MIF-related axes are shown as shared background interactions. **(D)** Candidate epithelial-to-T/NK interactions, highlighting relatively enriched MDK-related axes. **(E)** Candidate epithelial-to-fibroblast and epithelial-to-endothelial interactions. **(F)** Dot plot of key ligands and receptors in sender and receiver populations. All interactions are exploratory expression-based candidates rather than validated signaling mechanisms.

Balanced pooled CellChat analysis suggested a broader candidate outgoing communication profile from IFN-RS-high epithelial cells. The high state yielded 71 permutation-supported candidate ligand-receptor pairs with a cumulative supported probability of 1.270, compared with 60 pairs and 1.065 for the low state (Figure 6A). By receiver population, high versus low values were 22 pairs/0.390 versus 16/0.371 for myeloid cells, 17/0.303 versus 15/0.241 for T/NK cells, 15/0.308 versus 13/0.243 for fibroblasts, and 17/0.269 versus 16/0.209 for endothelial cells. These pooled differences were descriptive and were not treated as patient-level statistical effects.

Using a common ligand-receptor universe and retaining raw probability estimates in both states, the largest reproducible positive differences involved MDK-SDC1 toward T/NK cells (0.0814 versus 0.0656; delta 0.0158), MDK-ITGA6/ITGB1 toward endothelial cells (0.0683 versus 0.0549; delta 0.0134), and MDK-SDC2 toward fibroblasts (0.0539 versus 0.0432; delta 0.0107) (Figures 6B,D,E). By contrast, MIF-CD74/CXCR4, MIF-CD74/CD44, and PPIA-BSG showed near-zero high-minus-low differences and were interpreted as shared background communication axes (Figures 6C,E). In 20 balanced resampling iterations, the three selected MDK axes retained positive differences in all 20 runs. Ligand and receptor expression plots confirmed transcript availability in the relevant sender and receiver compartments (Figure 6F), but the inferred interactions remained candidate relationships requiring experimental validation.

### 3.7 IFN-RS-high epithelial cells show coordinated immune-effector and immune-regulatory transcriptional features

**FIGURE 7:**
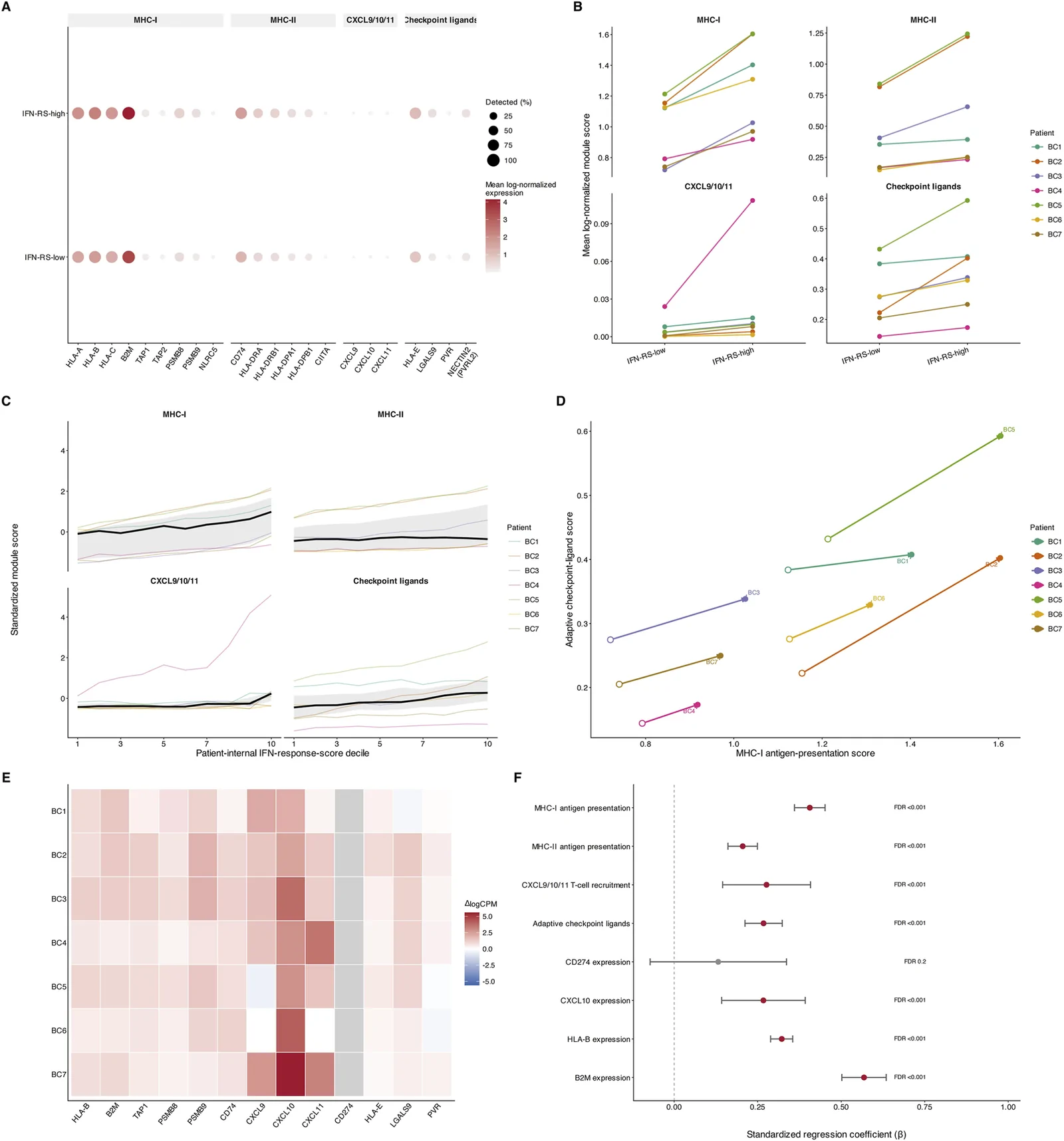
Immune-effector and immune-regulatory ligand features of IFN-response-score-high epithelial cells. **(A)** Dot plot showing MHC-I and MHC-II antigen-presentation genes, CXCL9/10/11 T-cell-recruitment-related chemokines, and immune-regulatory ligands in IFN-RS-low and IFN-RS-high cells. Dot size indicates detection percentage and color indicates mean log-normalized expression; cell counts were not used for inferential testing. **(B)** Patient-paired comparisons of four immune-feature module scores. IFI27, ISG15, and IFI6 were excluded from all modules. **(C)** Continuous relationships between patient-internal IFN-response-score deciles and standardized module scores. Thin lines represent individual patients, the thick line represents the median trend, and the ribbon shows the interpatient interquartile range. **(D)** Patient-paired arrow plot showing coordinated changes in MHC-I antigen-presentation and immune-regulatory ligand scores. **(E)** Heatmap of patient-specific pseudobulk high-minus-low differences; gray denotes unavailable stable estimates and not zero effect. **(F)** Mixed-effects model summary using 70 patient-by-decile aggregates with patient as a random intercept. Standardized coefficients, 95% confidence intervals, and BH-adjusted P values are shown.

IFN-RS-high epithelial cells showed higher detection and mean expression of multiple antigen-processing and presentation genes, CXCL9/10/11-related chemokines, and selected immune-regulatory ligands (Figure 7A). Examples included HLA-B detection increasing from 89.08% to 94.71%, PSMB9 from 21.57% to 40.25%, CD74 from 72.06% to 81.39%, HLA-DRA from 41.48% to 50.60%, CXCL10 from 1.02% to 2.77%, CXCL11 from 0.36% to 1.25%, HLA-E from 72.38% to 80.36%, LGALS9 from 24.33% to 37.67%, and NECTIN2/PVRL2 from 30.43% to 36.39%. CD274 was sparsely detected, with a maximum state-specific detection rate of 0.510%, and was not forced into the main dot plot.

All four patient-level module scores were higher in IFN-RS-high than IFN-RS-low cells in seven of seven patients (Figure 7B). Median high-minus-low differences were 0.280 for MHC-I antigen presentation, 0.100 for MHC-II-related antigen presentation, 0.00677 for CXCL9/10/11 T-cell-recruitment-related chemokines, and 0.0535 for immune-regulatory ligands. Each exact paired Wilcoxon test yielded P = 0.0156 and BH-FDR = 0.0156, the minimum attainable two-sided exact value for seven consistently positive nonzero pairs.

Module scores generally increased across patient-internal IFN-response deciles, supporting a continuous rather than threshold-limited relationship (Figure 7C). In paired two-dimensional analyses, all seven tumors moved toward higher MHC-I and higher immune-regulatory ligand scores from IFN-RS-low to IFN-RS-high states (Figure 7D). Patient-pseudobulk effects were consistently positive for HLA-B, B2M, TAP1, PSMB8, PSMB9, CD74, CXCL10, and HLA-E, whereas CXCL9, CXCL11, LGALS9, and PVR showed greater patient heterogeneity (Figure 7E).

Mixed-effects models fitted to 70 patient-by-decile aggregates showed positive standardized associations for MHC-I score (β = 0.406, 95% CI 0.360–0.452, FDR <0.001), MHC-II score (β = 0.205, 95% CI 0.161–0.249, FDR <0.001), CXCL9/10/11-related score (β = 0.277, 95% CI 0.145–0.408, FDR <0.001), immune-regulatory ligand score (β = 0.268, 95% CI 0.212–0.323, FDR <0.001), CXCL10 expression (β = 0.267, 95% CI 0.142–0.392, FDR <0.001), HLA-B expression (β = 0.322, 95% CI 0.289–0.355, FDR <0.001), and B2M expression (β = 0.568, 95% CI 0.502–0.635, FDR <0.001) (Figure 7F). CD274 did not show a stable continuous association (β = 0.132, 95% CI -0.072 to 0.336, FDR = 0.200). Thus, the immune-regulatory phenotype could not be reduced to a PD-L1-specific conclusion.

### 3.8 Bulk-transcriptomic corroboration in TCGA-BLCA

**FIGURE 8:**
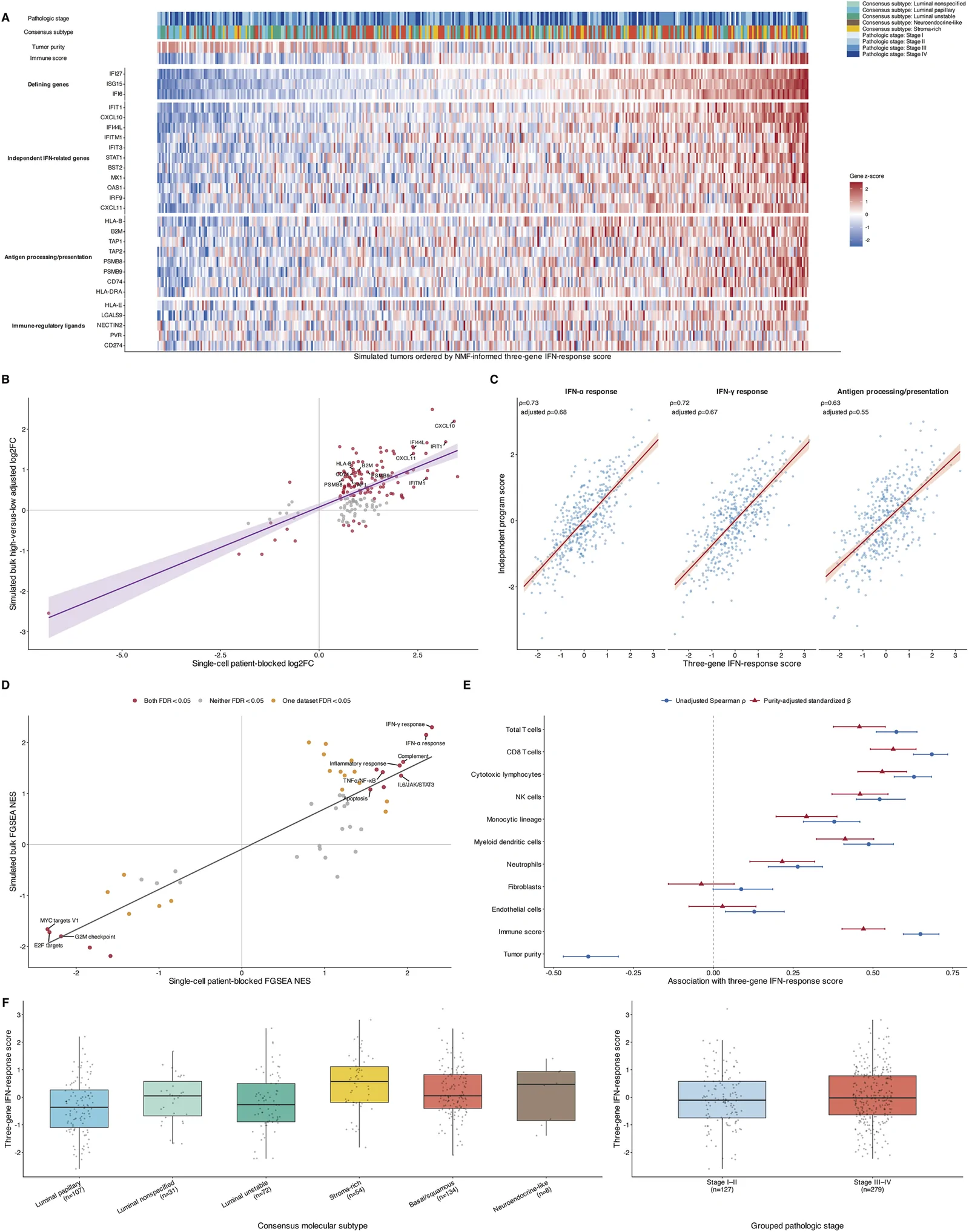
Bulk-transcriptomic corroboration and immune-context associations of the IFN-response program in TCGA-BLCA. **(A)** Heatmap of TCGA-BLCA primary tumor samples ordered by the NMF-informed three-gene IFN-response score, defined as the mean of sample-wise standardized expression values for IFI27, ISG15, and IFI6. Defining genes, independent interferon-response genes, antigen-processing and presentation genes, and immune-regulatory ligands are displayed in separate blocks. Available pathologic stage, consensus molecular subtype, tumor-purity estimate, and immune score are annotated above the heatmap. **(B)** Concordance between patient-blocked single-cell log2 fold changes and tumor-purity-adjusted TCGA-BLCA high-versus-low expression effects for shared genes. IFI27, ISG15, and IFI6 were excluded from the concordance analysis. **(C)** Associations of the three-gene IFN-response score with independent Hallmark interferon-gamma response, Hallmark interferon-alpha response, and Reactome antigen-processing and presentation scores after removal of the three defining genes. Crude and tumor-purity-adjusted correlations are shown. **(D)** Concordance between single-cell and TCGA-BLCA FGSEA normalized enrichment scores across shared Hallmark pathways. **(E)** Crude correlations and tumor-purity-adjusted associations between the three-gene IFN-response score and immune or stromal features estimated from TCGA-BLCA bulk transcriptomes. Error bars indicate 95% confidence intervals. **(F)** Distribution of the three-gene IFN-response score across consensus molecular subtypes and grouped pathologic stages. Clinical-outcome associations were evaluated using predefined continuous-score models and are reported according to the observed results.

In TCGA-BLCA primary tumors, ordering samples by the NMF-informed three-gene IFN-response score revealed coordinated gradients in independent interferon-response genes, antigen-processing and presentation genes, and selected immune-regulatory ligands (Figure 8A). Because the defining genes necessarily tracked with the score, corroboration focused on genes and pathways not used in score construction. After adjustment for tumor purity, gene-level effects showed positive concordance with the patient-blocked single-cell effects for shared genes, including IFIT1, CXCL10, IFI44L, IFITM1, HLA-B, B2M, TAP1, PSMB8, PSMB9, and CXCL11 (Figure 8B).

The continuous three-gene score correlated positively with independent Hallmark interferon-gamma, Hallmark interferon-alpha, and Reactome antigen-processing and presentation scores after removal of IFI27, ISG15, and IFI6; these associations persisted after tumor-purity adjustment (Figure 8C). Hallmark pathway effects also showed cross-platform directional concordance, with interferon, inflammatory, IL6-JAK-STAT3, TNF/NF-kappaB, complement, and apoptosis pathways aligned positively and proliferative programs aligned negatively (Figure 8D).

Bulk immune and stromal associations were partly attenuated by tumor-purity adjustment, indicating that the TCGA score captured both tumor-cell-intrinsic and microenvironmental contributions (Figure 8E). The score varied across consensus molecular subtypes and grouped pathologic stages (Figure 8F). Clinical-outcome models were interpreted as exploratory and did not provide a basis for presenting the score as an established independent prognostic biomarker. Overall, the strongest TCGA evidence was molecular corroboration of the IFN and antigen-presentation context rather than proof of epithelial origin or clinical utility.

### 3.9 Irradiation is associated with induction of the IFN-response transcriptional program in T24 cells

**FIGURE 9:**
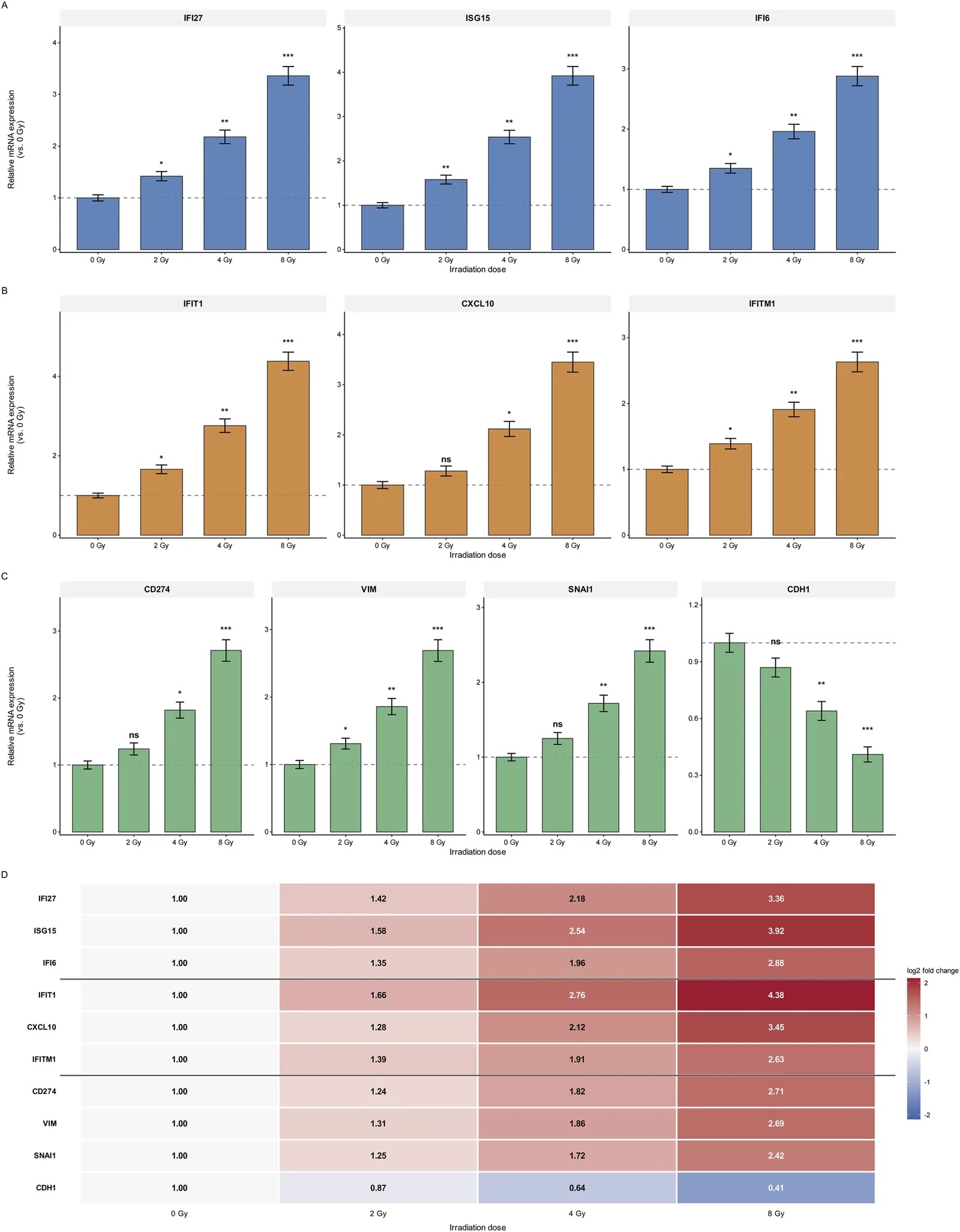
Irradiation-associated transcriptional responses of interferon-, immune-regulatory-, and EMT-related genes in T24 bladder cancer cells. Cells were exposed to 0, 2, 4, or 8 Gy and harvested 24 h later. **(A)** Relative mRNA expression of IFI27, ISG15, and IFI6. **(B)** Relative expression of independent interferon-associated genes IFIT1, CXCL10, and IFITM1. **(C)** Irradiation-associated changes in CD274, VIM, SNAI1, and CDH1 .**(D)** Heatmap summarizing mean dose-associated expression changes across all measured genes. Relative expression was calculated by the 2^−ΔΔCT^ method with 0 Gy as reference; statistical testing was performed on ΔCt values. Individual biological replicates and mean ± SEM are shown; n = 3 independent experiments.

qRT-PCR performed 24 h after irradiation showed dose-associated increases in the three defining IFN-response genes (Figure 9A). Relative to 0 Gy, IFI27 expression was 1.42-, 2.18-, and 3.36-fold at 2, 4, and 8 Gy; ISG15 was 1.58-, 2.54-, and 3.92-fold; and IFI6 was 1.35-, 1.96-, and 2.88-fold, respectively. The strongest responses occurred at 8 Gy, while lower doses produced smaller gene-specific effects.

Independent genes not used to define the single-cell score also responded to irradiation (Figure 9B). IFIT1 increased to 1.66-, 2.76-, and 4.38-fold; CXCL10 to 1.28-, 2.12-, and 3.45-fold; and IFITM1 to 1.39-, 1.91-, and 2.63-fold at 2, 4, and 8 Gy, respectively. CXCL10 showed limited change at 2 Gy but a clearer increase at four and 8 Gy, whereas IFIT1 showed the largest overall induction.

Auxiliary immune-regulatory and EMT-related transcripts showed coordinated but distinct changes (Figure 9C). CD274 increased from 1.00 at baseline to 1.24, 1.82, and 2.71; VIM to 1.31, 1.86, and 2.69; and SNAI1 to 1.25, 1.72, and 2.42 across increasing doses. CDH1 decreased to 0.87, 0.64, and 0.41. The combined heatmap summarized a dominant dose-associated increase in IFN-response genes, accompanied by secondary checkpoint- and EMT-related transcriptional changes (Figure 9D). These mRNA data supported irradiation responsiveness of the measured program but did not establish protein-level signaling, immune-cell recruitment, EMT completion, or radioresistance.

## 4 Discussion

This study defines a reproducible IFI27-centered interferon-response state within identity-gated bladder tumor epithelial cells. The principal evidence was not a single-cell P value or a single highly expressing tumor. Rather, it arose from repeated NMF across ranks and seeds, patient-internal state definition, patient-blocked pseudobulk modeling, cross-patient directional consistency, leave-one-patient-out stability, independent pathway scoring, and bulk-transcriptomic corroboration. Across these analyses, the state was characterized by IFIT1, CXCL10, IFI44L, IFI6, ISG15, HLA-B, B2M, CD74, and related antigen-processing genes, together with broad interferon, inflammatory, IL6-JAK-STAT3, TNF/NF-kappaB, and chemokine-response enrichment.

The findings add an epithelial-cell perspective to the increasingly complex bladder cancer single-cell landscape. Previous studies have described inflammatory fibroblasts, heterogeneous malignant epithelial lineages, diverse myeloid states, and interferon-dependent stromal programs in bladder cancer (Lai et al., 2021; Chen et al., 2020; Ma et al., 2022; Sfakianos et al., 2020). Our results show that interferon-response activity is also distributed across tumor epithelial subclusters and cannot be reduced to one patient-invariant cluster. The strong patient specificity of epithelial clusters, combined with substantial differences in absolute IFN score, emphasizes why patient-blocked analysis was essential. Methods that ignore biological replication can produce markedly inflated differential-expression results in single-cell data (Squair et al., 2021). Here, the reproducibility of the core state despite patient heterogeneity was more informative than uniform abundance across tumors.

The immune-feature analysis suggests a dual transcriptional context. IFN-RS-high epithelial cells showed coordinated increases in MHC-I processing, MHC-II-related genes, CXCL9/10/11-related chemokines, and selected immune-regulatory ligands. Interferon-driven gene-expression profiles in human tumors commonly combine antigen presentation and chemokine expression with adaptive regulatory features (Benci et al., 2016; Ayers et al., 2017). Tumor-cell MHC-II expression has also been associated with distinct immunologic phenotypes and response or resistance patterns in other cancers (Johnson et al., 2016; Johnson et al., 2018). In the present dataset, however, these signals were defined at the RNA level in identity-gated epithelial cells and should not be interpreted as proof that the cells function as professional antigen-presenting cells. Additional spatial colocalization, protein measurement, and functional antigen-presentation assays would be required.

Importantly, the immune-regulatory phenotype was not synonymous with PD-L1. CD274 was sparsely detected in untreated tumor epithelial cells and did not show a significant continuous association with the IFN-response score. By contrast, HLA-E, LGALS9, and NECTIN2 contributed more consistently to the broader ligand module. This distinction is biologically relevant because persistent interferon signaling can support multigenic regulatory programs beyond PD-L1 (Benci et al., 2016). It also prevents a common overinterpretation in which any interferon-associated state is assumed to be a PD-L1-high immune-evasion state. The transcriptomic state identified here is better described as immune-visible and immune-regulatory, with the balance between these components unresolved at the functional level.

The negative enrichment of E2F, G2M checkpoint, and MYC target programs suggests that IFN-RS-high cells had lower proliferative transcriptional activity. This observation is consistent with the growth-restrictive and stress-associated effects that interferon signaling can exert, but expression data alone cannot establish slower proliferation. Similarly, the positive apoptosis signature does not demonstrate cell death. These pathway results should be viewed as state descriptors that may guide future functional experiments rather than direct measurements of phenotype.

Balanced CellChat analysis nominated MDK-related interactions as relatively enriched candidates, particularly MDK-SDC1 toward T/NK cells, MDK-SDC2 toward fibroblasts, and MDK-ITGA6/ITGB1 toward endothelial cells. The common ligand-receptor universe and repeated downsampling reduced one source of bias and showed that these differences were more reproducible than MIF or PPIA axes, which behaved as shared background communications. Nevertheless, CellChat infers potential signaling from expression and curated interaction knowledge (Jin et al., 2021). The receiver populations were small, especially fibroblasts and endothelial cells, and patient-specific networks were not independently estimated. The MDK axes therefore represent prioritization targets for spatial or coculture validation, not evidence of functional intercellular signaling.

TCGA-BLCA corroboration extended the state beyond the discovery dataset. The three-gene score tracked with independent interferon and antigen-presentation genes, and gene- and pathway-level effects were directionally concordant with the patient-blocked single-cell results. Adjustment for tumor purity was important because bulk interferon signals can reflect both malignant cells and infiltrating immune cells. The persistence of several associations after purity adjustment supports, but does not prove, a tumor-intrinsic component. Variation across consensus molecular subtypes is also plausible given the known differences in immune and stromal composition among bladder cancer classes (Robertson et al., 2017; Kamoun et al., 2020). The present analyses did not establish the score as a prognostic or predictive biomarker; such a claim would require treatment-annotated cohorts and independent external validation.

The irradiation experiment provided a limited but useful bridge to treatment response. Ionizing radiation can activate tumor-cell nucleic-acid sensing and interferon pathways, and radiation-associated CXCL10 induction has been observed in experimental models (Demaria et al., 2016; Sharabi et al., 2015; Vanpouille-Box et al., 2017; Yang et al., 2021; Wang et al., 2023). In T24 cells, irradiation increased the three score-defining genes and independent IFIT1, CXCL10, and IFITM1 transcripts, indicating that the measured program was not restricted to untreated clinical specimens. The concurrent increase in CD274, VIM, and SNAI1 and decrease in CDH1 showed that irradiation produced a broader stress-associated transcriptional response. However, these observations were made at a single time point in 1 cell line and at the mRNA level. They do not demonstrate endogenous IFN secretion, receptor activation, immune-cell recruitment, EMT completion, or altered clonogenic survival.

The study has several limitations. First, the single-cell cohort contained seven tumors and one adjacent normal specimen; tumor-versus-normal composition was therefore descriptive. Second, the clinical specimens were treatment naive and lacked radiotherapy outcomes, so the IFN-RS-high state cannot be labeled a radiation-induced state in patients. Third, epithelial identity was established by transcriptomic gating rather than histologic localization or copy-number-based malignant-cell assignment. Fourth, IFN-RS-high and IFN-RS-low were relative patient-internal states and do not imply measured cytokine concentrations. Fifth, pooled communication analysis could not support patient-level inference. Sixth, TCGA bulk data cannot localize the signal to epithelial cells. Finally, the qRT-PCR experiment used three biological replicates in a single cell line and did not include protein, spatial, or functional validation.

Despite these limitations, the analysis provides a coherent framework for prioritizing follow-up studies. Spatial transcriptomics or multiplex RNA/protein imaging could determine whether IFN-RS-high epithelial cells colocalize with specific immune or stromal populations. Organoid or coculture models could test whether the MDK-related candidate axes alter lymphocyte, fibroblast, or endothelial behavior. Longitudinal sampling before and after radiotherapy or immune checkpoint blockade would be particularly valuable because interferon programs can support both immune recognition and adaptive resistance. Clinical trials of pembrolizumab, atezolizumab, and neoadjuvant checkpoint blockade have established the relevance of immune context in urothelial cancer (Bellmunt et al., 2017; Powles et al., 2018; Necchi et al., 2018; Powles et al., 2019), but the predictive value of this epithelial state remains an open question.

## 5 Conclusion

Multi-patient single-cell analysis identified an IFI27-centered interferon-response epithelial state in bladder cancer that was reproducible across NMF settings and robust to patient exclusion. The state was accompanied by coordinated antigen-processing, chemokine-related, inflammatory, and selected immune-regulatory transcriptional features, while CD274 itself remained sparse and inconsistent. Bulk-transcriptomic analyses corroborated the broader molecular context, and balanced pooled communication analysis prioritized MDK-related candidate interactions. Irradiation-associated qRT-PCR changes further showed that defining and independent components of the program were transcriptionally responsive in T24 cells. The results establish a rigorously characterized, hypothesis-generating epithelial state, but functional immune activity, clinical predictive value, and causal communication mechanisms require prospective validation.

## Data Availability

The datasets presented in this study can be found in online repositories. The names of the repository/repositories and accession number(s) can be found in the article/supplementary material.
